# Weakening Pin Bone Attachment in Fish Fillets Using High-Intensity Focused Ultrasound

**DOI:** 10.3390/foods6090082

**Published:** 2017-09-18

**Authors:** Martin H. Skjelvareid, Svein Kristian Stormo, Kristín Anna Þórarinsdóttir, Karsten Heia

**Affiliations:** 1 Department of Seafood Industry, Nofima AS, P.O. Box 6122, 9291 Tromsø, Norway; svein.stormo@nofima.no (S.K.S.); karsten.heia@nofima.no (K.H.); 2 Marel Fish Processing, Austurhraun 9, 210 Gardabaer, Iceland; Kristin.Thorarinsdottir@marel.com

**Keywords:** fish fillet, pin bones, HIFU, high intensity focused ultrasound, cod, salmon

## Abstract

High Intensity Focused Ultrasound (HIFU) can be used for the localized heating of biological tissue through the conversion of sound waves into heat. Although originally developed for human medicine, HIFU may also be used to weaken the attachment of pin bones in fish fillets to enable easier removal of such bones. This was shown in the present study, where a series of experiments were performed on HIFU phantoms and fillets of cod and salmon. In thin objects such as fish fillets, the heat is mainly dissipated at the surfaces. However, bones inside the fillet absorb ultrasound energy more efficiently than the surrounding tissue, resulting in a “self-focusing” heating of the bones. Salmon skin was found to effectively block the ultrasound, resulting in a significantly lower heating effect in fillets with skin. Cod skin partly blocked the ultrasound, but only to a small degree, enabling HIFU treatment through the skin. The treatment of fillets to reduce the pin bone attachment yielded an average reduction in the required pulling force by 50% in cod fillets with skin, with little muscle denaturation, and 72% in skinned fillets, with significant muscle denaturation. Salmon fillets were treated from the muscle side of the fillet to circumvent the need for penetration through skin. The treatment resulted in a 30% reduction in the peak pulling force and 10% reduction in the total pulling work, with a slight denaturation of the fillet surface.

## 1. Introduction

Fish fillets are made by cutting away the muscle on each side of the central backbone of the fish and trimming away the fins. In many fish species, so-called pin bones remain in the flesh after filleting and have to be removed to produce the desired boneless product. In most cases, pin bone removal is still a manual process, making it costly and time-consuming. In this paper, we consider the pin bones of two commercially important species, Atlantic cod (Gadus Morhua) and Atlantic salmon (Salmo salar), and study high-intensity focused ultrasound as a possible technology for weakening the attachment of the pin bones to the surrounding muscle. This could potentially enable automated pin bone removal and thereby improve fish processing efficiency.

**Figure 1:**
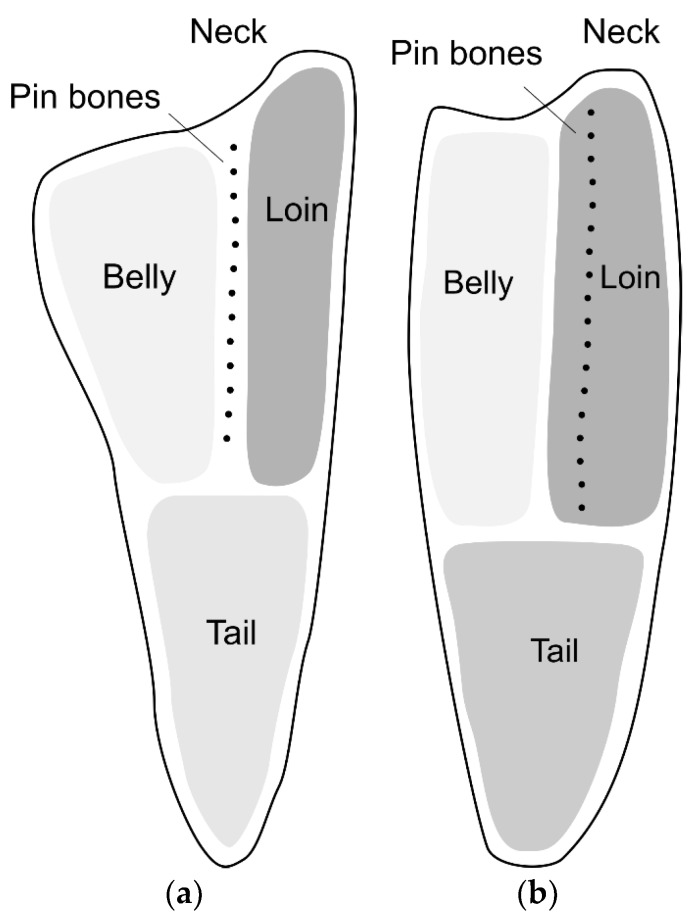
Schematic drawing of fish fillets showing the position of the pin bones. (**a**) Cod fillet. (**b**) Salmon fillet.

Fish muscle consists of a number of segments, called myomeres, separated by layers of connective tissue (CT), called myosepta. The smaller muscle segments converge at a thick layer of CT called the horizontal septum, dividing the muscles on the back of the fish (epaxial muscles) from the muscles on the belly side (hypaxial muscles). The horizontal septum can further be described as an array of tendons connecting the backbone to the skin [1]. In cod, the pin bones are embedded in this lattice of tendons in the horizontal septum, whereas in salmon, the pin bones are embedded in the epaxial muscles [2,3]. From a fish processing perspective, this means that for cod, the pin bones are positioned between the loin and belly parts of the fillet, while for salmon, the pin bones are positioned within the loin part. This is illustrated in Figure 1. The typical number of pin bones per fillet is approximately 17 for cod [2] and 30 for salmon [4,5].

Salmon also differs from cod in terms of the chemical composition. It is a fatty fish species, meaning that lipids are stored within the musculature, mainly in the CT between muscle segments [6]. Thus, the microstructure of the CT is different, which may influence the strength of bone attachments [7].

These anatomical differences between the two species may partly explain why higher forces are needed to pull out the bones from cod compared to salmon [2,4,5]. In both species, the strength of the pin bones’ attachment to surrounding tissues is reduced by post mortem ageing of the fillet. This mainly occurs though enzymatic activity in the muscle, which causes mild degradation of the CT [7].

Usually, the pin bones in cod fillets are removed by cutting out a strip containing the bones. In salmon, however, since the pin bones are located within the high-value loin part of the fillet, the bones are usually pulled out. This minimizes weight loss from the loin and ensures a visually appealing intact fillet. For salmon, bone removal is normally done after rigor mortis when bone attachments are partially degraded [7], as the risk of bone breakage and failure in bone removal is lower than in pre-rigor fish.

The CT of fish is heat-labile and denatures at lower temperatures than CT in products from land animals [8]. The major constituents of the CT are proteoglycans and fibrous proteins, with collagen being the most abundant protein [7]. The thermal properties of collagen and other muscle proteins have been studied by differential scanning calorimetry. The thermal transition of cod collagen appears as a small peak in the endothermic heat flow at approximately 30 °C and a stronger peak at approximately 40 °C [9]. In comparison, the thermal transition of salmon collagen appears as a single strong peak in the range of approximately 43–46 °C [10]. These findings indicate that mild heating might weaken the attachments of pin bones to the muscle. However, heating the whole fillet would result in a very poor product and the heating therefore needs to be limited to the area directly surrounding the bone. The main goal of the current work is to study whether high-intensity focused ultrasound can be used to perform such localized heating inside the fillet.

High-intensity focused ultrasound is a technique initially developed for cancer treatment in humans [11]. An ultrasound transducer creates a focused sound field with a very high intensity and, when the sound is directed into biological tissue, viscous losses cause part of the ultrasonic energy to be converted into heat. Because the sound field can be focused into a very narrow beam, the technique enables localized heat treatment inside the tissue. The focal point of an HIFU transducer has an ultrasonic intensity in the order of 1000 W/cm^3^, and tissue can be heated to >70 °C in 1–3 s [12]. The region of high temperature around the focal point of the transducer typically has a “cigar” shape, which is longer along the direction of wave propagation.

Biological tissues such as fish muscle are non-transparent, and the effects of HIFU treatment can therefore be hard to visualize. For this reason, experiments on HIFU “phantoms” are included in this paper. A phantom is a homogenous gel or solid that mimics the properties of biological tissue. Most methods for creating HIFU phantoms are based on fixing some kind of protein in a transparent gel [13,14,15]. When heated, the protein denatures and causes the treated area to appear white and opaque. This enables a direct visualization of the shape and size of the treated area.

The work described in this paper consists of three main parts: (1) A study of various parameters related to HIFU treatment, performed using HIFU phantoms; (2) a study of the heating effect on fish muscle samples, employing thermal imaging; and (3) a study of HIFU treatment on bones in fillets and its effect on bone attachment in the muscle.

## 2. Materials and Methods

### 2.1. HIFU Instrumentation

A Tektronix AFG2021 signal generator and an E&I 1040L amplifier were used to drive the HIFU transducers. Two different HIFU transducers manufactured by Precision Acoustics were used. The transducer specifications are listed in Table 1. The maximum input power to the transducers for continuous operation was established through practical tests and discussions with the manufacturer.

The transducers were operated at the maximum continuous input power in all experiments. The reason for this was twofold: Firstly, at low powers, the HIFU heating effect is low compared to the rate of heat dissipation in the rest of the sample, and thus the temperature never rises above a relatively low level; Secondly, the experimental conditions should give some insight into the potential for high-speed treatment on an industrial scale.

Note that although the 680 kHz transducer is operated at a higher power (120 W) than the 1 MHz transducer (100 W), the conversion of energy from sound waves to heat is generally more efficient at higher frequencies [12]. The transducers are therefore expected to yield approximately the same heating effect.

**Figure 2:**
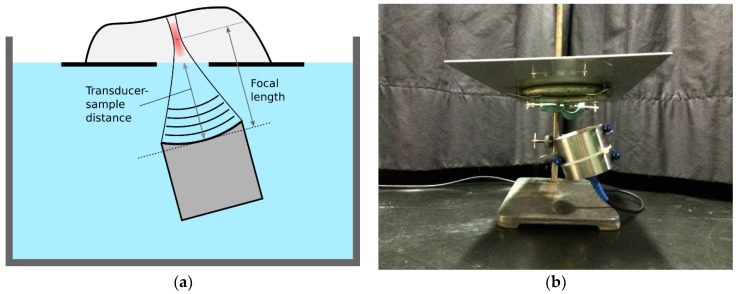
HIFU setup with a transducer and plate to hold the fillet. (**a**) Schematic illustration with a transducer placed in a water bath; (**b**) Image showing a transducer and plate mounted to a metal stand.

**Figure 3:**
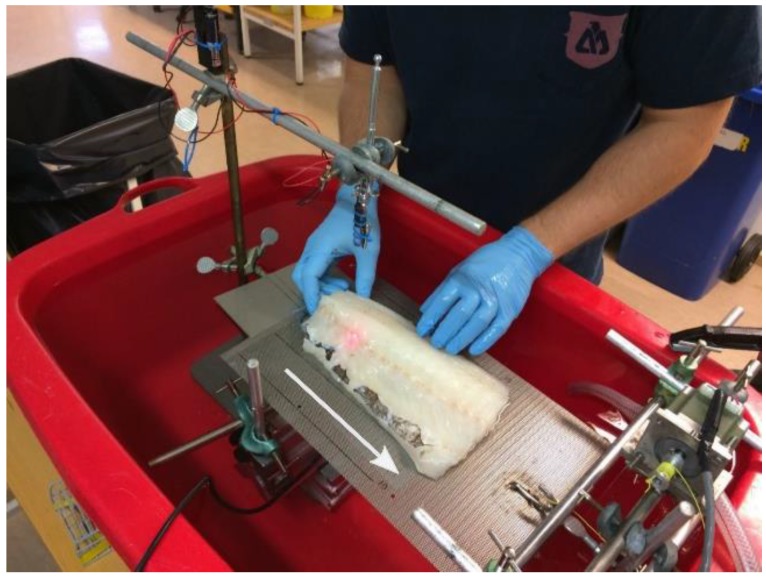
Complete setup placed in a plastic tub for immersion in water. The fish fillet in placed on a perforated plate with a slit in the middle to allow the ultrasound to pass through to the fillet. The plate is pulled by a stepper motor in the direction of the white arrow. A laser pointer mounted above the fillet indicates the position of the transducer focal point, enabling the operator to aim the ultrasound at the bone row during continuous treatment.

The treatment setup is shown in Figure 2. The transducer was placed in a water bath, pointing towards the water surface. The sample to be treated was placed on a plate, and the plate was placed over the transducer at the water surface. A hole in the plate allowed the ultrasound to travel from the water, directly into the sample. Figure 3 shows the complete setup placed in a plastic tub (used for submersion in water). A laser pointer was placed above the sample, pointing towards the focal point of the transducer. In this way, it was possible to see which part of the sample was being treated. For continuous treatment along a line, the sample was placed on a second plate and moved across the transducer beam, as indicated by the white arrow in Figure 3. The second plate was pulled by a stepper motor with an adjustable speed, and the position of the object under treatment was adjusted by hand during pulling, to keep the row of bones in the focal zone.

### 2.2. Ultrasound Phantoms

One common method for making HIFU phantoms is to use bovine serum albumin (BSA) as the protein and mix it with a polyacrylamide gel [13]. However, the polyacrylamide gel is made using acrylamide, which is a known neurotoxin. With some experimentation, we found that a standard agar gel could be mixed with a BSA solution to form a physically stable and relatively transparent phantom. The agar gel is completely non-toxic and was therefore preferred over the polyacrylamide. This approach is similar to that described in [16], for the fabrication of thermal therapy phantoms.

The method for making phantoms was to dissolve the BSA and agarose in separate solutions, carefully mixing them, pouring the mix into a container, and then letting it set in a refrigerator to generate a solid form. Because the agarose needs to be dissolved at high temperatures (close to 100 °C), the BSA was dissolved in a separate solution at room temperature, to avoid premature denaturation of the protein.

Initial experiments were conducted to establish how much BSA could be dissolved in water, and 23.1% was found to be an approximate limit. An agarose concentration of 4.3% was found to be suitable—as high as possible without gelling during the mixing of the two solutions. The temperature of the solutions was brought to approximately 30 °C (BSA) and 70 °C (agarose), and the solutions were carefully combined in a 60/40% mixture (BSA/agarose). This resulted in a final concentration of 13.8% BSA and 1.72% agarose in the phantoms.

To study the interaction between ultrasound and fish bones, phantoms with implanted pin bones were made. The bones were first collected from a number of cod fillets and sorted according to size. The phantom container was then partly filled with BSA/agar mixture, which was allowed to set for a few minutes, and the bones were placed on the sticky surface of the partly solidified gel. Lastly, the container was completely filled with the BSA/agar mixture, leaving the bones in the middle of the finished phantom.

### 2.3. Fish Samples

Cod for experiments was sourced from the Tromsø Aquaculture Research Station. The cod was caught in 2014, fed at the facility for approximately one and a half years, and slaughtered on 23 October 2015. The weight of the cod after gutting and heading was in the range 3.0–6.9 kg, with 75% of the fish in the 3.0–5.0 kg range. The cod was stored on ice for three days, filleted at Nofima, and stored for an additional day on ice before the experiments.

Whole salmon were purchased from Dragøy, a local fishmonger in Tromsø, and the details of the handling are therefore not known. The gutted weights of the salmon were in the 5–6 kg range. The salmon was filleted at Nofima on 3 November 2015, at which time it was in a state of rigor mortis. However, this did not seem to affect the condition of the finished fillet. The fillets were subsequently stored on ice for two days and were past the rigor state by the time of the experiment.

### 2.4. Thermal Imaging of Heating Effect

To study the HIFU heating effect in fish muscle tissue, samples of cod and salmon were first subjected to HIFU treatment and then imaged using a thermal camera. Loins from cod and salmon were cut to fit on the HIFU treatment plate and were treated at different transducer-sample distances (25–75 mm) using the 680 kHz and 1 MHz transducer. Cod and salmon samples were treated for 15 and 20 s, respectively. A slightly higher treatment time was used for salmon, as initial tests indicated that the heating effect was lower in salmon. Experiments were conducted with both skinned and unskinned fillets. After treatment, the loin was sliced along the treated area, perpendicular to the fillet surface. The slice was subsequently imaged using a Testo 870 thermal camera, yielding information on the temperature distribution as a function of the lateral position and depth inside the fillet. Each image was taken 15 s after the end of treatment.

### 2.5. HIFU Treatment Protocol for Bone Pulling Experiments

In the experiment, to study the effect of HIFU treatment on the bone pulling force, cod fillets were treated with the 1 MHz transducer and salmon fillets with the 680 kHz transducer, as the initial experiments suggested that these had the best heating effect for the respective species. The transducer-fillet distance was set to 50 mm, based on initial experiments that showed that for cod fillets, the best bone heating effect was obtained with distances in the range of 45–55 mm (for salmon, initial experiments showed no obvious optimal distance). The transducer was tilted approximately 15 degrees to align the transducer beam with the direction of the bones in the fillets.

Cod fillets were treated from the skin side, with the muscle side facing upwards, enabling easy tracking of the bone position. Salmon fillets were treated from the muscle (opposite) side, since initial experiments indicated that skin effectively blocks the ultrasound.

Cod fillets were divided into groups with and without skin, with eight fish (16 fillets) in each group. Each group was treated at a speed of 1.5 mm/s. The 10 salmon which were purchased for the experiment were intended for a single group with skin, treated at a speed of 1.5 mm/s. However, after treating three fillets, it became apparent that the treatment was heating the surface to such a degree that it began to split up and fall apart. Consequently, the speed was adjusted to 3 mm/s for the remaining seven fillets.

In each experiment series, one of the fillets (right or left) was used as the control, and the other was treated with HIFU. The left and right fillets were alternated for the treatment and control, to avoid any possible systematic differences between left and right fillets.

### 2.6. Bone Pulling

The bones were pulled using a Stable Micro Systems TA-HDi texture analyzer with a 25 kg load cell. The bones were gripped with artery forceps and pulled vertically upwards at 10 mm/s (maximum speed), whilst the fillet was held down by hand. The force time series from the texture analyzer were analyzed to calculate the peak force during pulling and the integrated force (work) from the start to end of the pulling. During the experiments, it was observed that cod bones would generally loosen after the peak value was reached, while salmon bones would detach from the muscle in a short series of tugs.

For cod, 10–12 bones were pulled, starting at the bone closest to the tail, and for salmon, 12 bones were pulled, starting with bone number 4 from the tail (bones 1–3 were very small and therefore not included).

## 3. Results

### 3.1. Phantom Experiment Results

#### 3.1.1. Distance between Transducer and Sample—Boneless Phantom

An experiment was performed to investigate how the distance between the transducer and the sample affects the treatment. A 30 mm thick phantom was treated with a 1 MHz transducer for 15 s, with the transducer-sample distance adjusted between 40 and 80 mm in 5 mm steps. The phantom was at room temperature, approximately 20 °C.

**Figure 4:**
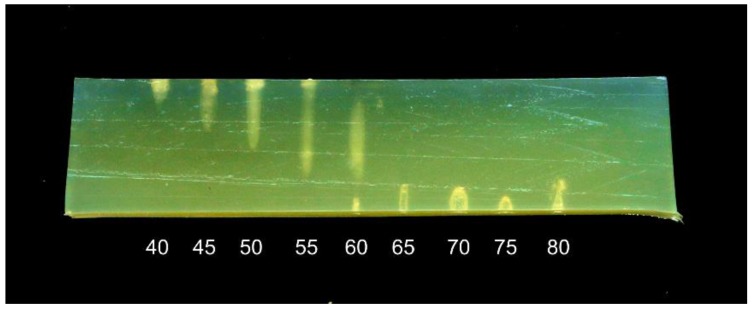
Phantom treated with a 1 MHz transducer for 15 s at distances between 40 and 80 mm (distance marked below each treatment position).The transducer was placed below the phantom.

The results are shown in Figure 4. When the transducer is close to the sample, most of the heat is dissipated in the upper surface of the phantom, with denaturation causing a bright spot and a depression in the surface. With increasing distance, the denatured area extends further into the phantom, and at a 60 mm distance, when the focal point is centered in the phantom, the denatured area has a “cigar” shape. This shape corresponds to that observed in the application of HIFU in medicine, under ideal conditions [12]. However, at distances of 65 mm and above, there is no visible denaturation inside the phantom, but rather a series of small holes on the bottom surface of the phantom. When the focal point is close to the water-phantom interface, the sound field seems to have a kind of “hammering” effect rather than a heating effect.

The results illustrate that for a relatively thin sample, such as a fish fillet, the distance between the transducer and the sample is critical for the shape of the treated area and the intensity of the treatment.

#### 3.1.2. Treatment Time—Boneless Phantom

A second experiment was performed to study the effect of treatment time on the heating effect. A 30 mm thick phantom was treated with the 1 MHz transducer at a 55 mm distance, at points placed 10 mm apart. The treatment time was adjusted between 3 and 30 s. The phantom was at room temperature prior to treatment.

**Figure 5:**
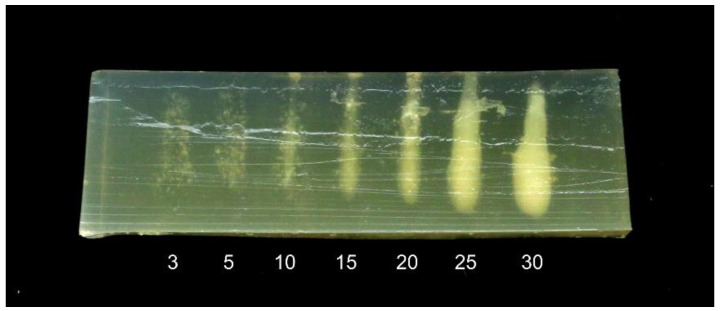
HIFU phantom treated with a 1 MHz transducer at 55 mm distance, for 3–30 s (treatment time marked below each treatment position). The phantom was at room temperature prior to treatment. The horizontal stripes in the image are due to imperfect cutting of the phantom slice.

The results are shown in Figure 5. For treatment times of 3–5 s, the treatment causes small specks to appear in the phantom. These may be due to denaturation close to pre-existing gas bubbles or ultrasound-induced cavitation bubbles [13]. For treatment times of 10 s and above, a cigar-shaped denatured area begins to form, growing thicker with longer treatment times. Note also that for 25 and 30 s, the denatured area is thicker at the bottom. This is consistent with observations from previous studies [12] showing that ultrasonic cavitation results in more heating in the area closest to the transducer. The results illustrate that not only the intensity but also the shape of the treated area is dependent on time.

#### 3.1.3. Sample Temperature—Boneless Phantom

In HIFU treatment, the treated area must be heated above a certain threshold temperature to cause denaturation. The input power and treatment time required to reach this threshold are dependent on the initial temperature of the sample. In fish processing plants the temperature of the product is kept close to 0 °C in order to minimize unwanted microbiological and enzymatic activity, and an experiment was conducted to study the effect of treatment at these low, more realistic temperatures. HIFU phantoms were cooled to approximately 4 °C and treated with the 1 MHz transducer at a 55 mm distance for treatment times between 3 and 50 s.

**Figure 6:**
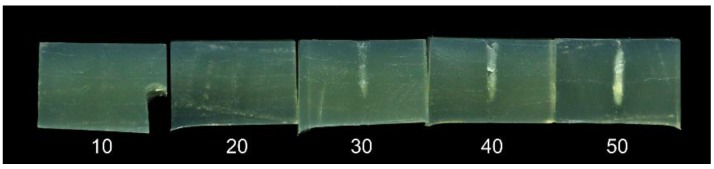
Slices of HIFU phantoms, treated with a 1 MHz transducer at a 55 mm distance for 10, 20, 30, 40, and 50 s (left to right). The phantom temperature was approx. 4 °C before treatment.

Images of the sliced phantoms are shown in Figure 6. For treatment times of 20 s or less, no denaturation is visible. However, for 30–50 s, a thin cigar-shaped denaturation area is clearly visible. Comparing these results to the results for the phantom at room temperature (Figure 5), it is clearly seen that the heating effect is significantly reduced by the lower sample temperature.

#### 3.1.4. Bone in Phantom

**Figure 7:**
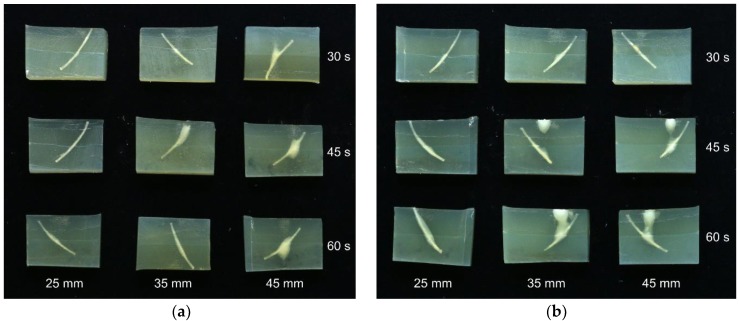
Slices of phantoms with embedded bones, treated at a 25–45 mm distance and for 30–60 s. The phantom temperature was approx. 4 °C before treatment. (**a**) Treated with a 680 kHz transducer; (**b**) Treated with a 1 MHz transducer.

A phantom experiment was performed to study the interaction between bones and the HIFU sound field. Phantoms of a 30 mm thickness with implanted bones were treated with the 680 kHz and 1 MHz transducers at distances of 25, 35, and 45 mm for 30, 45, and 60 s at each distance. The 25 mm distance was included in order to study the effect of defocusing the sound field. The phantoms were cooled to 4 °C prior to treatment. After treatment, the phantoms were sliced and imaged. The results are shown in Figure 7.

Generally, the heating effect at a 25 mm distance is lower than at 35 and 45 mm, due to the defocused nature of the sound field. However, with the 25 mm distance, the denaturation is only close to the bone, while for 35 and 45 mm, there are clouds of denaturation outside of the bone. For the 680 kHz transducer, the clouds are centered on the bone, while for the 1 MHz transducer, there is also an area of denaturation in the upper surface of the phantom. The result for the 1 MHz treatment at 25 mm for 60 s (lower left corner of Figure 7b) is especially interesting. In this case, there is a clearly visible area of denaturation along almost all of the bone, with no additional “clouds” outside of this. This result indicates that the use of a defocused sound field may be useful to reduce the amount of denaturation in areas apart from outside of the bone, while still yielding a heating effect close to the bone.

### 3.2. Salmon Experiment Results

#### 3.2.1. Effect of Transducer Distance and Presence of Skin

**Figure 8:**
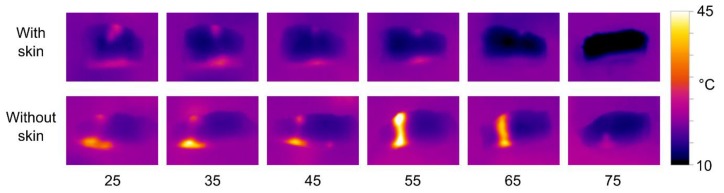
Thermal images of salmon samples treated with the 680 kHz transducer for 20 s. The transducer-sample distance is listed below each set of images.

The results of thermal imaging of HIFU treated salmon samples are shown in Figure 8. The experiment was performed with both the 680 kHz and the 1 MHz transducer, but to limit the number of figures, only the results for the 680 kHz transducer are shown here. The effects using the 1 MHz transducer were slightly weaker, but otherwise quite similar.

The thermal images clearly show that there is a significantly stronger heating effect in samples without skin compared to skinned fillets. The heat distribution is also affected by the transducer-sample distance, with short distances resulting in more heat dissipation at the upper surface, and longer distances resulting in more heat dissipating at the lower surface. However, the patterns of heat distribution are less pronounced than what was observed with the phantoms. There is a tendency for most of the heat to be dissipated at the lower surface, i.e., the side closest to the transducer. Using the 680 kHz transducer on skinned fillets, a distance of 55 mm produced significant heating throughout the samples.

To test how the fat layer underneath the skin affects the HIFU treatment, an additional test was performed on a salmon sample. Here, a salmon loin sample was prepared with three test areas; one with intact skin, one where skin was removed but with the fat and dark muscle underneath remaining, and one with the muscle directly exposed. Each area was treated with the 680 kHz transducer at a 55 mm distance for 20 s.

**Figure 9:**
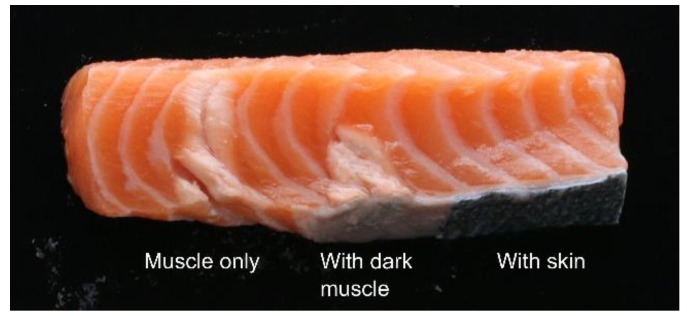
Salmon fillet treated with a 680 kHz transducer at a 55 mm distance for 20 s, in three different points (from left to right): Skinned, with skin removed but fat and dark muscle remaining, and with skin.

The result is shown in Figure 9. With the muscle directly exposed, the HIFU treatment yields an area of denaturation throughout the whole sample. However, with some fat and dark muscle remaining, the effect only reached halfway into the sample. In the part of the loin with the skin intact, no denaturation was observed. This indicates that for optimum efficiency of the treatment, both skin and fat/dark muscle should be removed.

#### 3.2.2. Bone Pulling Experiments

**Figure 10:**
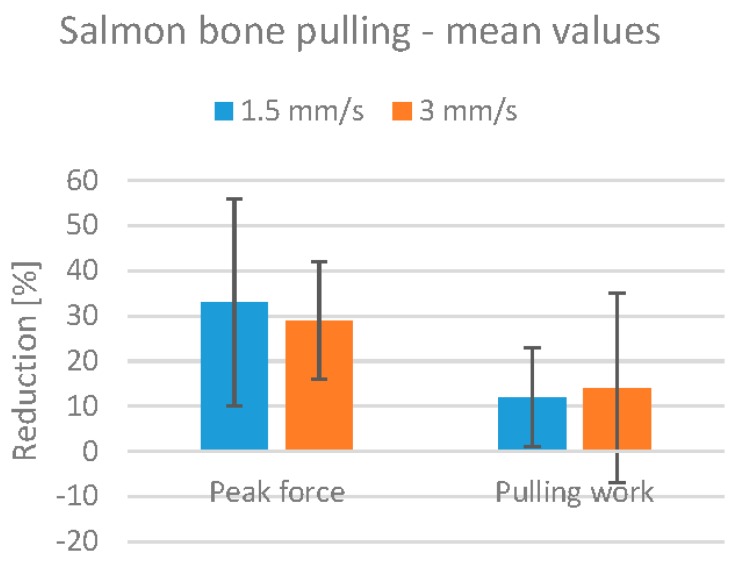
Mean values for the reduction of bone pulling force and work in salmon fillets, with error bars marking corresponding mean standard deviation values. Reduction percentages are calculated relative to the control fillet from the same fish.

The effect of HIFU treatment on bone pulling is shown in Figure 10, which shows the mean reduction in peak pulling force and work. The reduction in peak pulling force is approximately 30%, and the reduction in pulling work is approximately 12% for both treatment speeds. Note, however, that only three fillets were treated at 1.5 mm/s, and the results are therefore not as reliable as those for the 3.0 mm/s treatment.

There is a large difference between the peak pulling force and the pulling work values. During pulling, the salmon bones would detach from the fillet in a short series of tugs, rather than one single tug with a peak force value. The pulling work metric therefore seems more representative for salmon.

There was only one bone broken and no bones slipping in all of the salmon experiments. There was thus no problem of “overtreatment” in this series of experiments.

**Figure 11:**
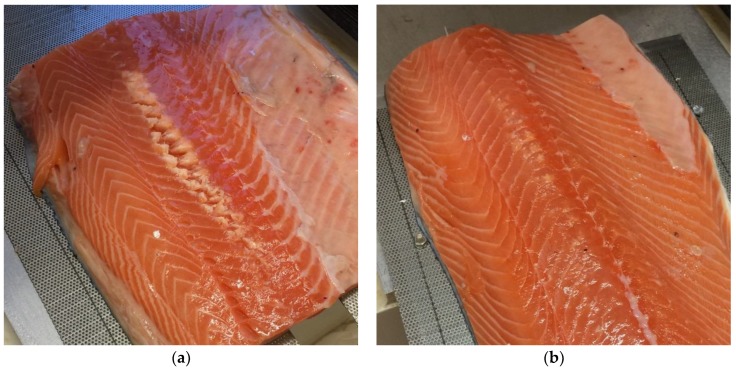
Examples of salmon fillets after HIFU treatment. (**a**) Treated at 1.5 mm/s. Denaturation and splitting clearly visible; (**b**) Treated at 3 mm/s. Some denaturation visible, no splitting.

The effect of HIFU treatment on the fillet surface was not systematically recorded, but the effect was generally more severe at treatment speeds of 1.5 mm/s. Example images of fillets after treatment are shown in Figure 11.

### 3.3. Cod Experiment Results

#### 3.3.1. Effect of Transducer Distance and Presence of Skin

**Figure 12:**
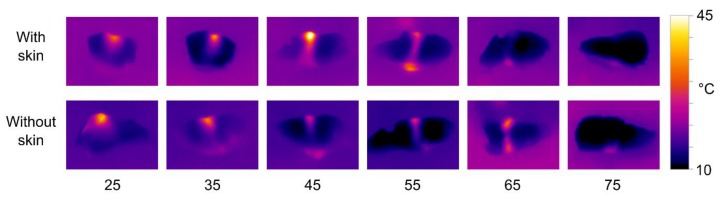
Thermal images of cod samples treated with the 1 MHz transducer for 15 s. The transducer-sample distance is listed below each set of images.

The results of thermal imaging of HIFU treated cod samples are shown in Figure 12. The experiment was performed with both the 680 kHz and the 1 MHz transducer, but to limit the number of figures, only the results for the 1 MHz transducer are shown here. The effects using the 680 MHz were slightly weaker, but otherwise quite similar (a tendency opposite to that of salmon).

As with the salmon, the effect of transducer-sample distance to the cod is similar to that observed in phantoms, but the effect is less pronounced. There is also a clear tendency for the heat to be mostly dissipated at the upper and lower surfaces of the samples. This is probably due to the transitions between media of different acoustic impedances (water, tissue, and air). There are no clearly visible differences between samples with or without skin in the thermal image.

Additional experiments were performed to study the heating effect on bones during continuous HIFU treatment along the bone row. Cod fillets with and without skin were treated with the 680 kHz and 1 MHz transducer, at distances of 45 and 55 mm, which seemed promising based on the thermal imaging results. The fillets were treated at a speed of 1 mm/s, and were cut along the row of bones after treatment.

**Figure 13:**
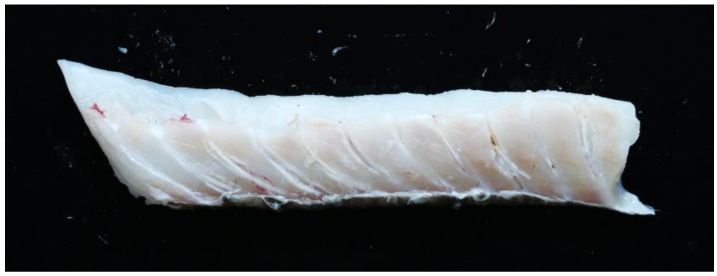
Example of a cod fillet treated with a 1 MHz transducer at a 45 mm distance, with continuous treatment along the row of bones at a speed of 1 mm/s. The fillet was cut along the row of bones to visualize the treatment effect. Note that some bones have become detached from the muscle, and that some have been “burned”, resulting in a brown color. The image represents a “best case” with regard to the treatment effect, and is not representative of all fillets treated.

The strongest heating effect was seen for the cod fillet with skin, treated with the 1 MHz transducer at a 45 mm distance. This fillet is shown in Figure 13. Many of the bones have clearly detached from the muscle, and there is also one bone which has become burned. However, the rest of the fish muscle does not have a very “cooked” appearance, indicating again that the bones absorb heat more efficiently that the muscle.

Note that similar experiments were conducted on salmon samples, but without any visible effect on the bones or the tissue surrounding the bone. Images from these experiments are therefore not included here.

#### 3.3.2. Bone Pulling Experiments

**Figure 14:**
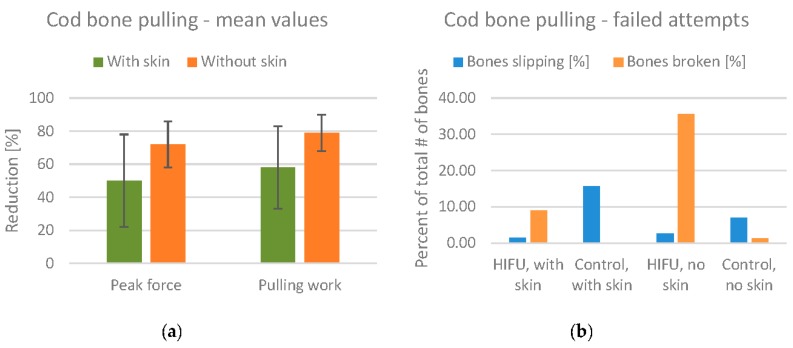
(**a**) Mean values for the reduction of bone pulling force and work in cod fillets, with error bars marking corresponding mean standard deviation values. Reduction percentages are calculated relative to the control fillet from the same fish; (**b**) Number of failed bone pulling attempts in cod fillet experiments.

The mean values for the reduction of pulling force/work are shown in Figure 14a. The mean reduction in the peak pulling force is 50% for fillets with skin, and 72% for fillets without skin. Note also that the standard deviation is lower for fillets without skin.

Although the results seem promising with regard to reducing the bone attachment, there were also a significant number of failed bone pulling attempts. These are divided into attempts where the bones broke during pulling (most often due to overheating) and attempts where the bone slipped from the artery forceps. These numbers are summarized in Figure 14b. For the HIFU treated fillets without skin, 35% of the bones broke during pulling, indicating that the fillets were overtreated. Studying the pulling force values in detail, we found that the number of broken bones was much higher in the smallest fillets. For the treated fillets with skin, the number of broken bones was only 9%. Thus, the presence of skin reduces the effect of HIFU treatment to some degree, but also reduces the number of broken bones. The number of bones slipping is included in the figure to show that this number is quite low for HIFU treated fillets compared to the control fillets.

**Figure 15:**
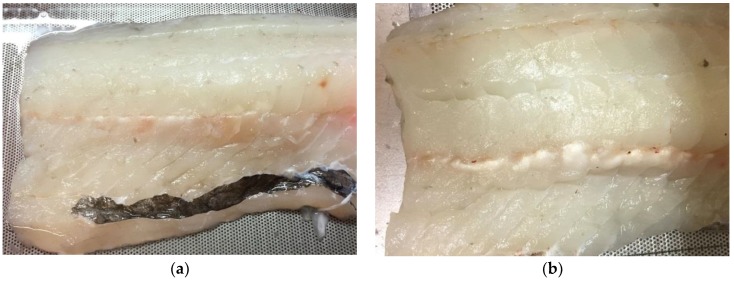
Example of a cod fillet after HIFU treatment. (**a**) Fillet with skin: A small amount of white denaturated muscle visible along the bone row; (**b**) Fillet without skin. Denaturation is clearly visible along the bone row, and some bones are burned.

The amount of visible denaturation on the fillets was not systematically recorded, but the amount of denaturation was generally higher for the fillets without skin. Example images of fillets after treatment are shown in Figure 15.

## 4. Discussion

For HIFU phantoms that are comparable to fish fillets in thickness (30 mm), correct adjustment of the transducer-sample distance was critical for efficient heating and a well-shaped heat distribution inside the samples. However, the experiments on fish samples did not show the same sensitivity to distance. The distance affected the distribution of heat, but most of the heat was either distributed at the upper or lower surfaces of the fillet. The treated areas also did not have the “cigar” shape seen in the phantoms. This illustrates that the behaviour of HIFU in fish fillets is very different than in its original application of cancer treatment in humans, where the “sample” is several centimetres thick. In thinner samples such as the fish fillets, the heat distribution is more difficult to control.

The experiments on phantoms also indicate that bones absorb ultrasound energy and convert it to heat more efficiently than muscle tissue. This effect is, to some degree, confirmed by the experiments on cod samples. The bones appeared to detach from the surrounding muscle, with only small denaturation effects in the muscle. This “self-focusing” effect for bones may be useful in an industrial application. Rather than targeting each bone in the fillet with high precision, it may be possible to apply a strong, non-focused sound field and rely on the bones to absorb the sound waves where needed. This could reduce the accuracy requirement for positioning of the transducer.

The experiments on salmon samples with and without skin showed that its skin (and the dark muscle and fat underneath) effectively blocks the ultrasound and thereby reduces the heating effect. From a salmon processing perspective, this limits the usefulness of HIFU treatment, as a large portion of salmon fillets are sold with skin. In the experiments, a possible “workaround” of treating the fillets from the muscle side was tested. Unfortunately, the results show that the reduction in the bone pulling force after HIFU treatment is relatively small. One possible explanation for this is that the heating effect along the bone row appeared not to penetrate beyond a few millimetres into the fillet. However, some of the results obtained for single salmon samples indicate that the HIFU effect can penetrate throughout the whole fillet. This discrepancy is not yet understood, and further studies on salmon are needed before definite conclusions can be drawn.

The initial experiments on single cod samples suggested that the presence of skin had no effect on the heat intensity and distribution in the samples. However, the bone pulling experiments showed that the heating effect was stronger for skinned fillets, resulting in a larger number of burned and broken bones. These unwanted effects were mainly seen for small bones, while larger bones were still strong enough to be pulled. This indicates that the problem could be alleviated by a better adjustment of the power of the HIFU treatment according to bone size (estimated from fish weight and bone position).

For practical reasons, all of the experiments were conducted “post-rigor”. In many cases, fish fillet producers would like to perform filleting and bone removal before rigor mortis to reduce the processing time and increase the product shelf life [17]. Future research should include experiments on such pre-rigor fillets to determine its usefulness in these types of samples.

The HIFU transducers were driven with 100–120 W of electrical input power, enabling the treatment of pin bone rows at 2–3 mm/s. Even assuming that 5 mm/s is sufficient, there is still a significant gap between this and industrial fish processing speeds, which are usually around 400 mm/s, i.e., 80 times faster. The industrial application of HIFU will therefore require custom made HIFU transducers with significantly higher power levels than any “off-the-shelf” transducers available today.

A future study should also consider the practical aspects of acoustic coupling between the transducer and the fish fillets. In the experiments described here, coupling was achieved by submerging the transducer in water, and placing the fillet in the surface of the water. This may be impractical in an industrial implementation, and alternative “dry” coupling methods [18] should be researched and tested.

The use of HIFU technology for localized heating may also be interesting in other areas of food processing. HIFU could potentially be used as a mild treatment to ease the deboning of products such as chicken or beef. Also, as a gastronomic technique, HIFU could, for example, be used to create dishes that are cooked inside while remaining raw on the outside. Future research may pave the way for such new applications.

## 5. Conclusions

We have studied high intensity focused ultrasound (HIFU) as a potential technology for weakening the attachment of pin bones in fish fillets. Experiments were conducted on cod and salmon samples, in addition to HIFU phantoms. The results show that the thin geometry of fish fillets causes the heat to be mainly dissipated at the surfaces of the fillets, making the effects of HIFU treatment less controllable than in thicker geometries (e.g., human patients). The pin bones are seen to absorb the ultrasound energy more efficiently than the surrounding tissue, creating a “self-focusing” effect which can possibly be exploited for bone heating, avoiding the need to aim the HIFU transducer with a high degree of accuracy. For salmon fillets, the skin partly blocks the ultrasound from entering the fish muscle, significantly reducing the heating effect. This effect is less pronounced for cod fillets with skin, allowing for the significant heating of fillets both with and without skin.

HIFU treatment was more effective for decreasing bone attachment in cod than in salmon. For cod, a reduction in pulling force of 50–70% was observed, but care must be taken to avoid the overtreatment of bones, which can result in bones breaking during pulling. For salmon, HIFU treatment resulted in a reduction of 30% for peak force and 10% for total pulling work. Thus, for the experimental setup tested here, HIFU treatment seems more promising for the processing of cod than for salmon. However, further experimentation and development is required to determine the full potential of HIFU treatment to aid in fish processing.

## Figures and Tables

**Table 1:** HIFU transducer specifications.

Serial Number	Operating Frequency (Hz)	Active Area Diameter (mm)	Focal Length (mm)	Width of Focal Zone (FWHM) (mm)	Max. Continuous Input Power (W)
PA439	680	60	75	4.3	120
PA500	1000	60	75	2.8	100
